# Resolution of Right Hemidiaphragm Paralysis following Cervical Foraminotomies

**DOI:** 10.1155/2018/6195179

**Published:** 2018-03-04

**Authors:** Neal Singleton, Matthew Bowman, David Bartle

**Affiliations:** Orthopaedic Department, Tauranga Hospital, Cameron Road, Tauranga, New Zealand

## Abstract

**Introduction:**

Hemidiaphragm paralysis secondary to phrenic nerve palsy is a well-recognised medical condition. There are few case reports in the literature documenting resolution of hemidiaphragm paralysis following cervical spine surgery. This case report documents our experience with one such case.

**Case Presentation:**

A 64-year-old man was referred to the orthopaedic service with right hemidiaphragm paralysis. He had a previous history of asbestos exposure and polio and was initially seen and investigated by the respiratory physicians. He also reported intermittent neck pain and an MRI scan showed right-sided cervical foraminal stenosis. He underwent posterior right C3/4 and C4/5 foraminotomies, and by three months postoperatively, his hemidiaphragm paralysis had resolved and his shortness of breath had also improved.

**Conclusion:**

This report documents a unique case of resolution of hemidiaphragm paralysis following posterior unilateral cervical foraminotomies.

## 1. Introduction

The phrenic nerves (C3/4/5) supply motor function to the hemidiaphragms. The motor supply of each hemidiaphragm comes purely from the phrenic nerves, and so conditions affecting the phrenic nerve or its nerve roots cause hemidiaphragm paralysis. Hemidiaphragm paralysis secondary to phrenic nerve palsy is a well-recognised medical condition with multiple causes. However, there are few case reports in the literature documenting resolution of hemidiaphragm paralysis following the cervical spine surgery. The cases that have previously been described involve patients with concomitant tetraparesis treated with spinal cord decompression with or without foraminotomies. This case report documents resolution of hemidiaphragm paralysis and improved respiratory function with unilateral cervical nerve root decompression alone, a finding that has not previously been described.

## 2. Case Report

**Figure 1:**
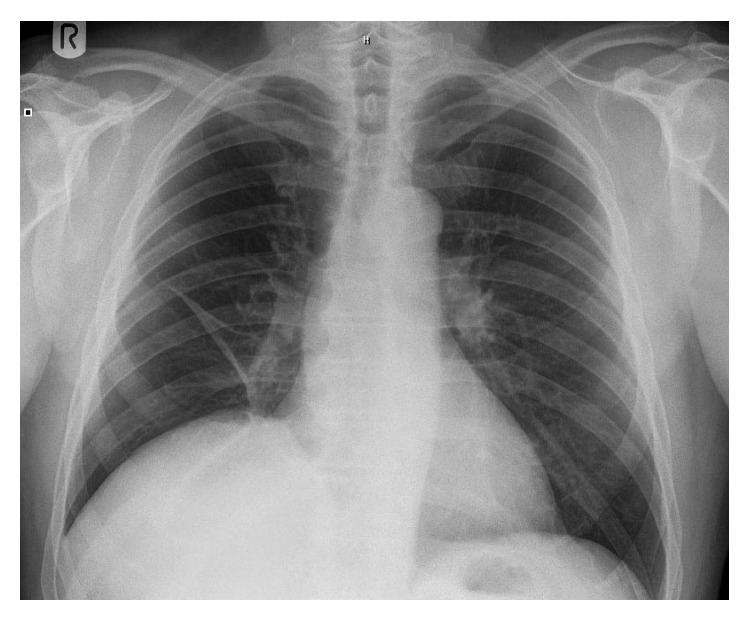
Preoperative chest radiograph in a 64-year-old man with right-sided stenosis at C3/4 and C4/5 demonstrating an elevated right hemidiaphragm.

**Figure 2:**
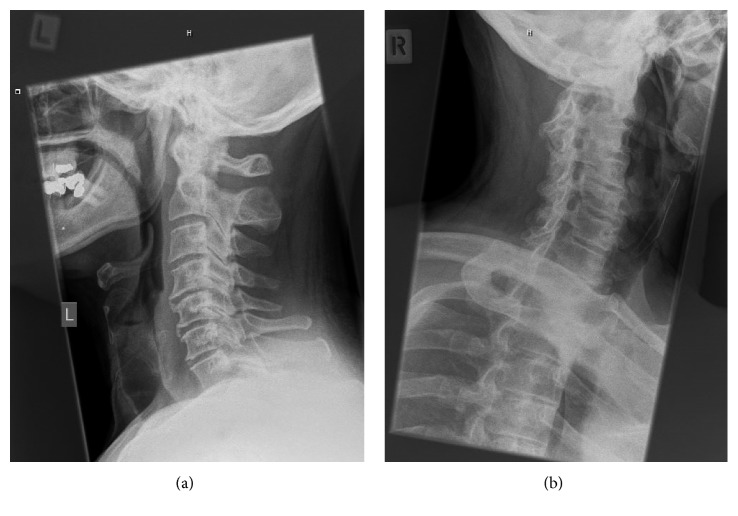
Preoperative AP and oblique cervical spine radiographs showing degenerative spondylosis.

**Figure 3:**
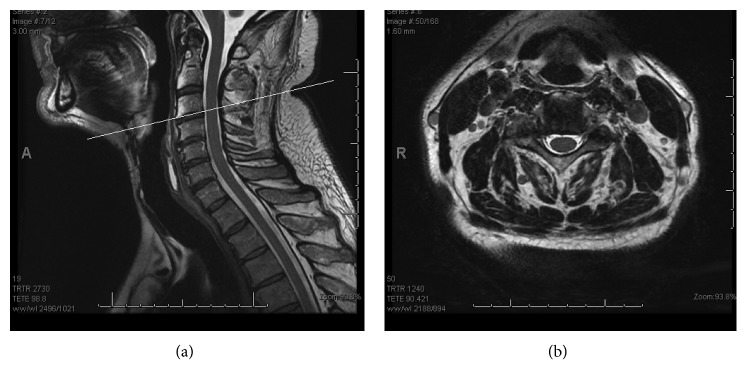
Preoperative T2-weighted sagittal MRI scan at the C3/4 level with corresponding axial sequence illustrating right-sided foraminal stenosis due to a combination of disc bulge, uncovertebral osteophyte, and facet joint hypertrophy.

A 64-year-old man was referred to the orthopaedic service with right hemidiaphragm paralysis. He had initially presented to his general practitioner reporting subjective shortness of breath after stand-up paddleboarding. His past medical history was significant for asbestos exposure and polio. Chest radiograph revealed an elevated right hemidiaphragm (Figures 1 and 2). A previous chest radiograph taken five years before showed normal diaphragmatic contours. He was subsequently referred to the respiratory physicians where workup included a chest CT scan (which revealed no intrathoracic abnormality) and dynamic fluoroscopic sniffing test (which confirmed complete right hemidiaphragm paralysis). He reported innocuous injuries to his neck in the past and intermittent neck pain for which he had previously consulted both a chiropractor and an osteopath. An MRI scan was undertaken which showed right-sided cervical foraminal stenosis (with uncovertebral and facet joint osteophytic changes at C3/4 and C4/5) (Figure 3). He was therefore referred to the orthopaedic service. On examination, he had no focal cervical spine tenderness with a well-preserved range of motion. He did have some generalized right shoulder girdle and upper limb wasting and weakness compared to the left (presumed to be secondary to his postpolio syndrome). His upper limb reflexes were intact and symmetrical with the contralateral side.

**Figure 4:**
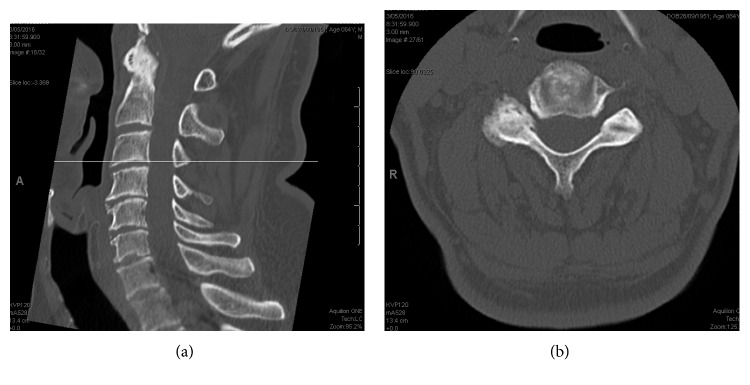
Preoperative CT scan of the cervical spine with sagittal slice and corresponding axial slice at the C3/4 level illustrating right-sided foraminal stenosis.

The underlying cause for his hemidiaphragm paralysis, whether it was related to his cervical foraminal stenosis or postpolio syndrome, was indeterminant (Figure 4).

**Figure 5:**
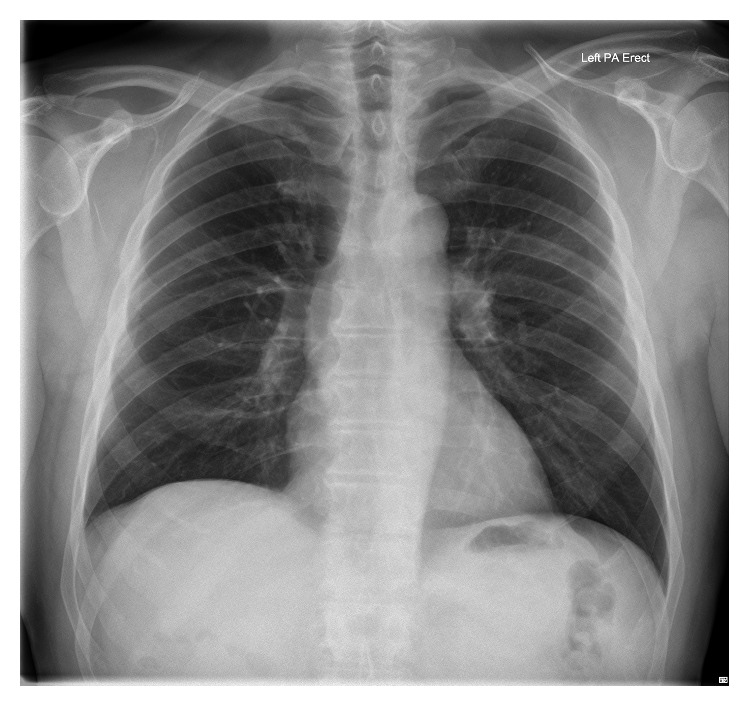
Chest radiograph three months postoperatively showing resolution of right hemidiaphragm paralysis.

**Figure 6:**
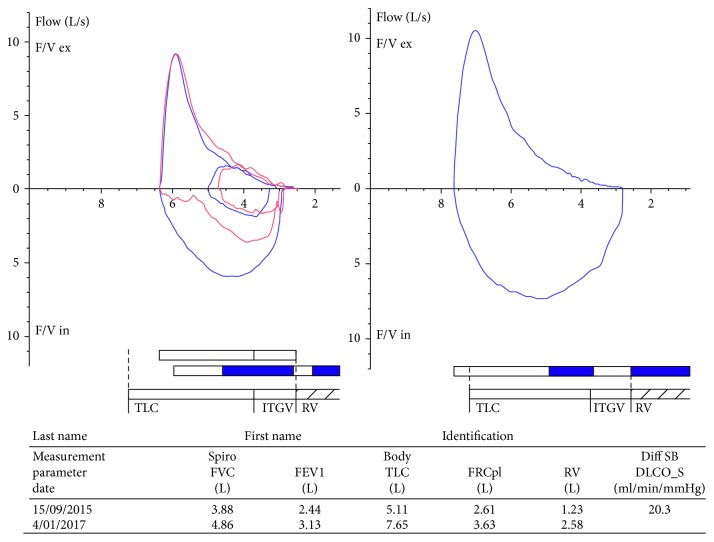
Comparison of preoperative and postoperative spirometry results showing marked improvement in respiratory function.

After obtaining multiple subspecialist opinions, a decision was made to proceed with posterior right C3/4 and C4/5 foraminotomies accepting that this may not have any effect on his shortness of breath. Surgery proceeded uneventfully as did postoperative recovery. By three months postoperatively, his hemidiaphragm paralysis had completely resolved on chest radiograph, and his shortness of breath had also improved (Figure 5). Comparison of preoperative and postoperative spirometry lung function showed significant improvements in all parameters tested: increases in FVC from 3.88L to 4.86L, FEV1 from 2.44L to 3.13L, TLC from 5.11L to 7.65L, FRCpl from 2.61 to 3.63, and RV from 1.23L to 2.58L. A graphic representation of these findings is shown in Figure 6. A satisfactory outcome was thus achieved.

## 3. Discussion

The phrenic nerve originates from cervical nerve roots C3–5 with the dominant supply coming from C4. The phrenic nerves are the sole motor supply to the hemidiaphragms and also provide proprioceptive fibres to the central part of each hemidiaphragm. Common causes of phrenic nerve palsy include idiopathic, malignancy (primary lung tumour or metastatic disease), trauma (penetrating injury and postsurgical, following central venous catheterisation and cervical manipulation), neuromuscular disease (polio or multiple sclerosis), inflammation (pneumonia or HSV), brachial plexus palsies, and direct compression (aortic aneurysm). However, the aetiology of diaphragmatic paralysis remains unidentified in more than two-thirds of patients [1].

It is possible that these idiopathic cases are caused by unidentified nerve root compression in the cervical spine. Poliomyelitis can result in the degeneration of the anterior horn cells, innervating both hemidiaphragms and the accessory respiratory muscles. Additional damage to the axons of the surviving anterior horn cells as a result of nerve root compression may result in clinically significant respiratory dysfunction as was evident in this patient.

Acute dyspnoea secondary to diaphragmatic paralysis can also occur following minor cervical trauma. Parke and Whalen described two patients with severe cervical myelopathy who developed respiratory insufficiency related to phrenic nerve palsy after cervical manipulation [2]. Merino-Ramirez et al. also reported on two patients who developed hemidiaphragm paralysis, one after chiropractic cervical manipulation and the other following a motorcycle accident [3].

Respiratory compromise is a known complication of acute cervical spinal cord injury but rarely is it considered in less acute settings such as in cases of degenerative cervical spondylosis.

There are few case reports in the literature documenting resolution of diaphragmatic paralysis due to cervical nerve root compression following cervical spine surgery. Hayashi et al. reported on a 64-year-old man with dyspnoea who had bilateral hemidiaphragm paralysis secondary to cervical spondylosis [4]. Following cervical laminoplasty, his diaphragm paralysis completely resolved, and his respiratory symptoms and spirometry also improved. Fregni et al. reported a case of phrenic nerve palsy in a 53-year-old with cervical spondylotic myelopathy [5]. Buszek et al. reported on a case of left hemidiaphragm paralysis with shortness of breath secondary to C3/4 neural foramen compression which resolved completely after laminectomy [6]. Rudrappa and Kokatnur reported on a 64-year-old man with acute shortness of breath and dyspnoea and an elevated left hemidiaphragm with severe cervical spondylosis on MRI [7]. Following cord decompression, his respiratory symptoms resolved. Yu et al. reported the case of an 82-year-old man who presented with respiratory symptoms [8]. He went on to have cardiac angiography and ultimately triple coronary artery bypass grafting which failed to improve his symptoms. He then developed generalised weakness, and an MRI showed C2–7 central canal stenosis and myelomalacia. Following laminectomy and instrumented fusion, his respiratory symptoms completely resolved. To our knowledge, this is the first case that shows resolution of hemidiaphragm paralysis and improved subjective and objective respiratory function after cervical foraminotomies alone.

There are numerous studies in the literature that show impaired respiratory function in patients presenting with cervical pathology. Ishibe and Takahashi compared 84 patients with cervical pathology with an age-matched control group of patients without cervical pathology and found that those in the cervical group had significantly lower respiratory function (vital capacity and percent forced vital capacity) [9]. Within the cervical group those with more cephalad pathology (C4 and cephalad) had more severe respiratory dysfunction. Postoperatively, those in the cephalad cervical group were shown to have significantly improved respiratory function. Similarly, Yanaka et al. reported on 12 patients with cervical myelopathy treated with laminoplasty [10]. Pre- and postoperative spirometry was performed, and it was found that tidal volume increased significantly.

## 4. Conclusion

Similar to other case reports in the literature, this report documents a case of resolution of right hemidiaphragm paralysis following C4 and C5 nerve root decompression via posterior cervical foraminotomies (C3/4 and C4/5 levels), a finding not previously described. Although rare, cervical nerve root compression as a cause of phrenic nerve palsy should be considered in patients presenting with hemidiaphragm paralysis and respiratory symptoms as surgical management can result in resolution of paralysis and potential improvement in respiratory symptoms.
